# Symmetrical Drug‐Related Intertriginous and Flexural Exanthema Following Amoxicillin‐Clavulanate in a Patient With Chronic Obstructive Pulmonary Disease

**DOI:** 10.1002/ccr3.71268

**Published:** 2025-10-14

**Authors:** Niranjan Pudasaini, Anil Kumar Singh Dangol, Surendra Shrestha, Aashutosh Pokharel, Aarif Khan

**Affiliations:** ^1^ Department of Dermatology Shree Birendra Hospital Kathmandu Nepal; ^2^ Nepalese Army Institute of Health Sciences Kathmandu Nepal

**Keywords:** amoxicillin, baboon syndrome, clavulanic acid, symmetrical drug‐related intertriginous and flexural exanthema

## Abstract

Symmetrical Drug‐Related Intertriginous and Flexural Exanthema is a rare and often overlooked cutaneous drug reaction. Prompt recognition of its symmetrical, flexural distribution and association with systemic drugs like amoxicillin‐clavulanate can prevent misdiagnosis and ensure timely management.

## 1 Introduction

Symmetrical Drug‐related intertriginous and flexural exanthema (SDRIFE) is a rare symmetrical erythematous rash that appears on the gluteal and intertriginous area after exposure to systemic drugs [1]. Amoxicillin‐clavulanate is a widely used antimicrobial combination that not only inhibits the synthesis of the bacterial cell wall but also enhances efficacy by inhibiting β‐lactamase enzymes, thereby overcoming resistance [2]. We report a classical case of SDRIFE in a 68‐year‐old female following administration of amoxicillin‐clavulanate. This case report has been reported in line with the CARE guideline [3].

## 2 Case History

**FIGURE 1:**
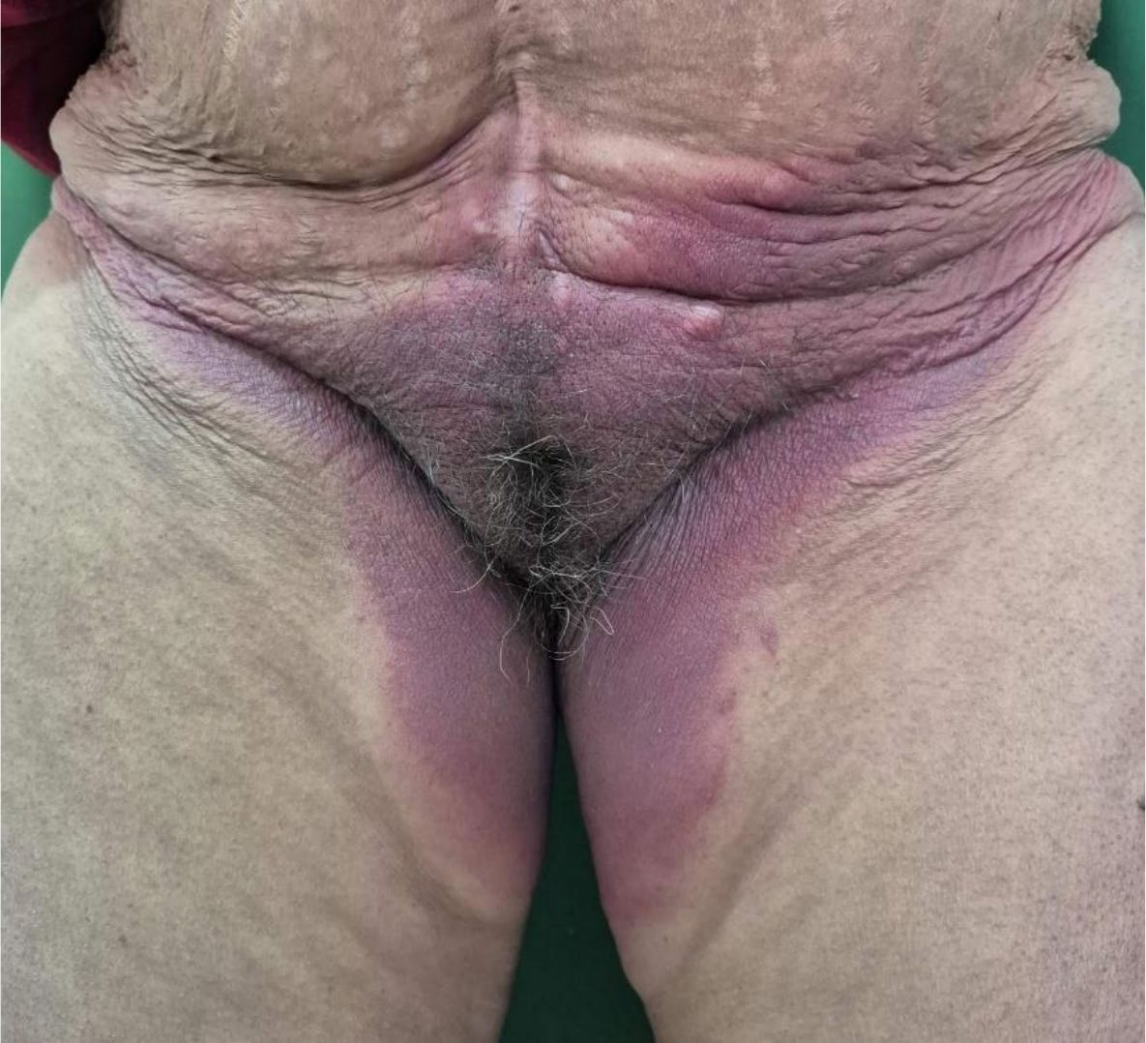
Erythematous rash on the inguinal region.

**FIGURE 2:**
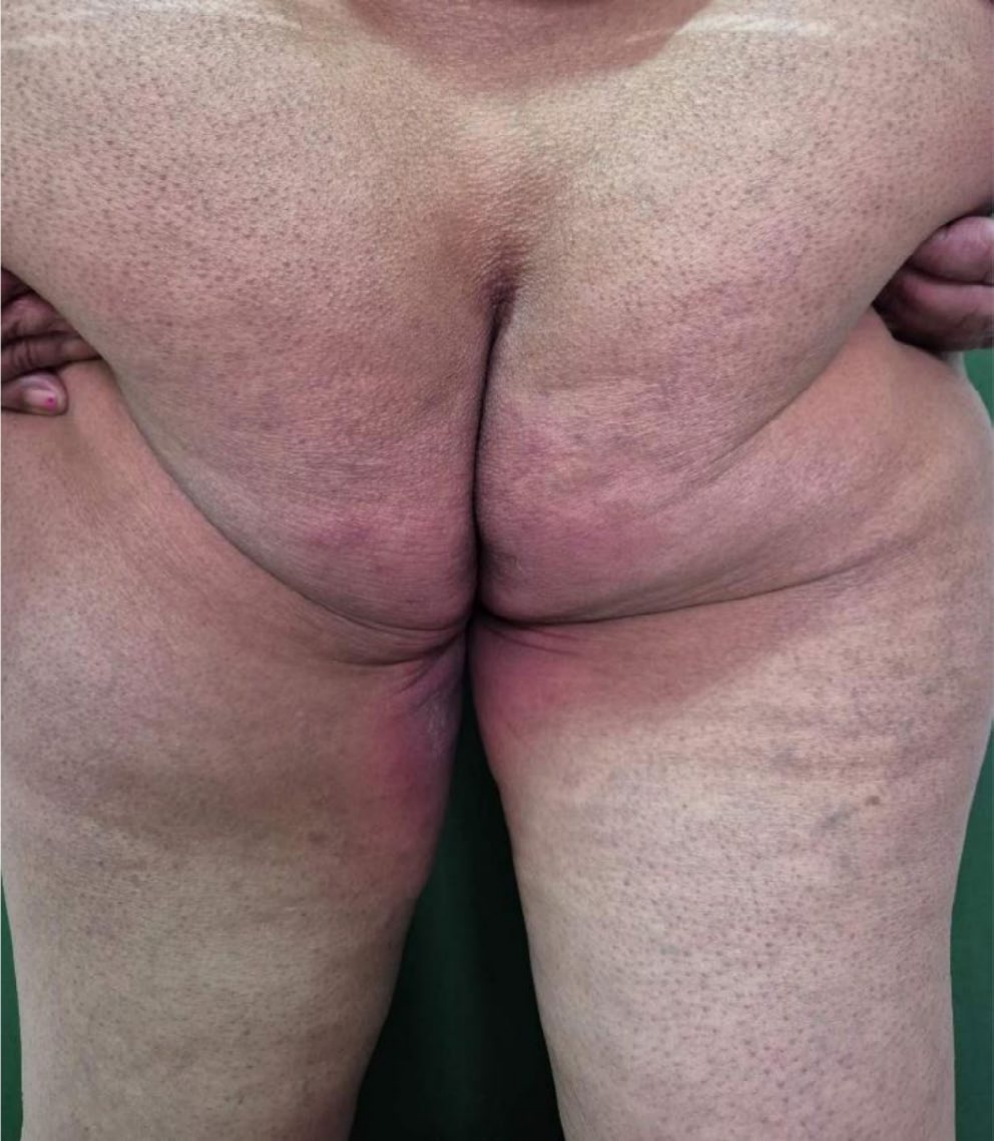
Erythematous rash on the gluteal region.

**FIGURE 3:**
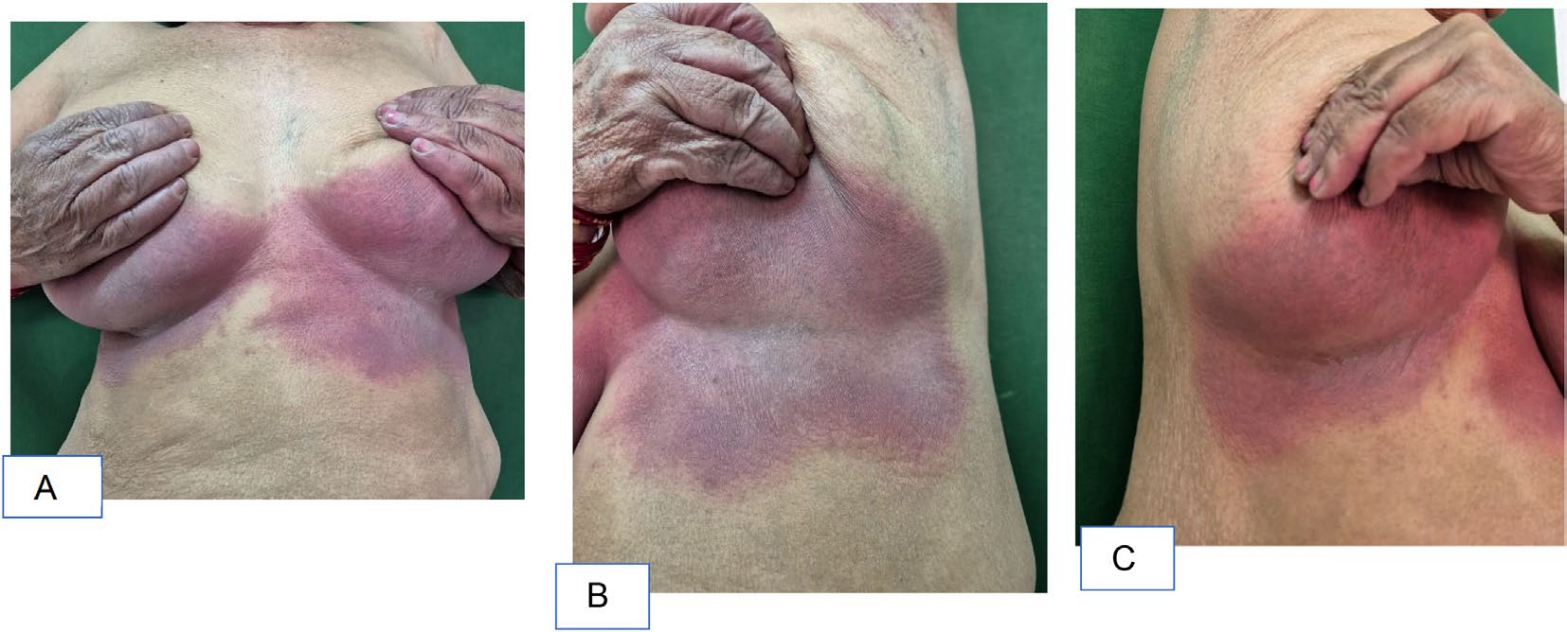
(A) Frontal view of an erythematous rash on the inframammary folds, (B) Left lateral view of erythematous rash on inframammary folds, (C) Right lateral view of erythematous rash on the inframammary folds.

**FIGURE 4:**
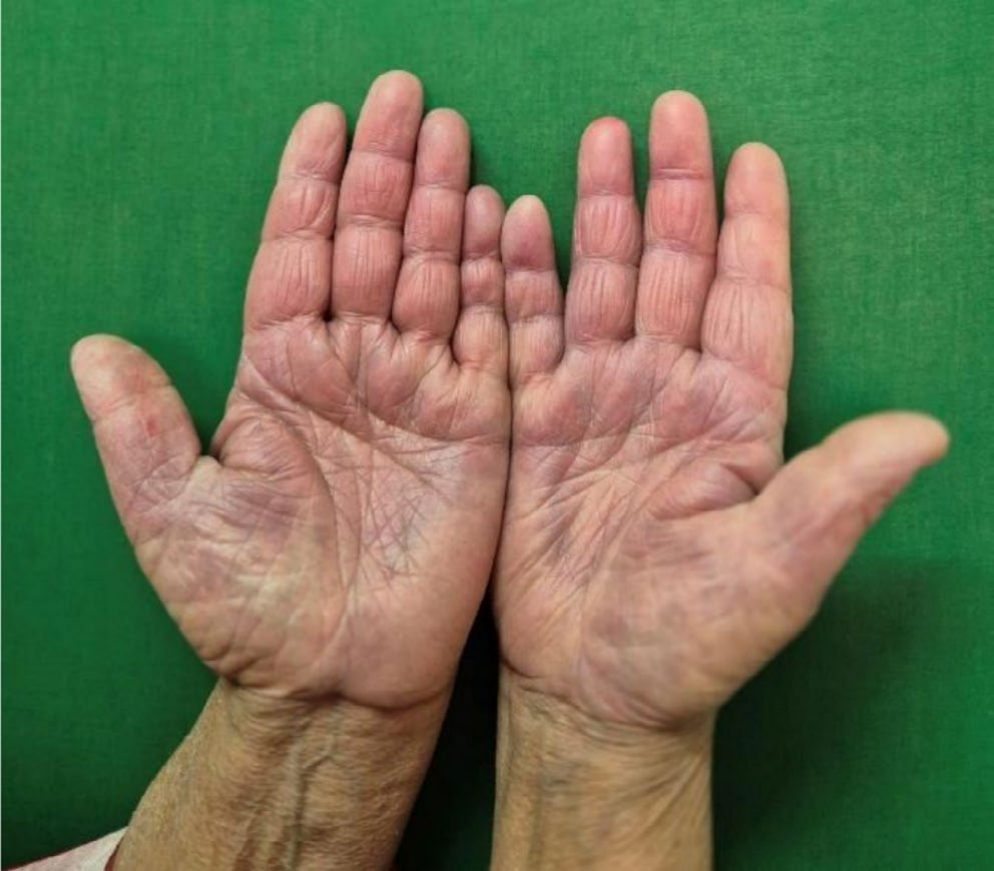
Erythematous rash on the palmar surface of the hand.

**FIGURE 5:**
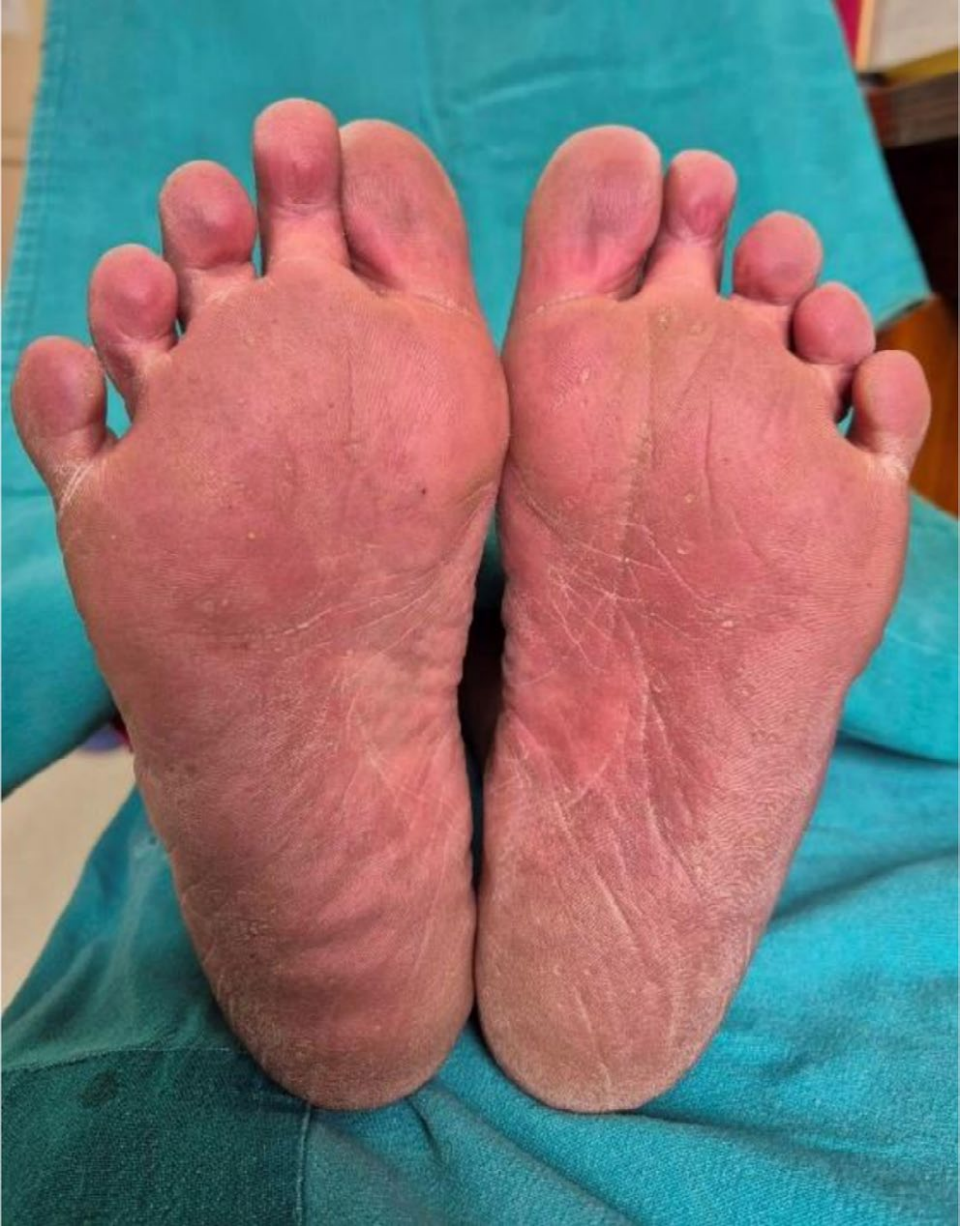
Erythematous rash on the plantar surface of the foot.

A 68‐year‐old female who is a known case of chronic obstructive pulmonary disease (COPD) presented to the dermatology outpatient department with complaints of erythematous rashes that developed within 24 h of initiating the culprit drug. The rashes were symmetrically distributed, involving the gluteal region, inframammary folds, palms, soles, and other intertriginous areas as shown in Figures 1, 2, 3, 4, 5. She reported mild pruritus and a burning sensation without mucosal involvement or systemic symptoms.

Five days before the onset of the rash, the patient had visited the emergency department with shortness of breath, productive cough, and fever. On examination, she was dyspneic, with bilateral wheeze noted on auscultation. A diagnosis of acute exacerbation of COPD was made, and she was discharged on oral amoxicillin‐clavulanate (clavum) along with Tab Azithromycin, Tab Prednisolone, Tab Lasilactone, Syrup Ambrodril, Ipravent Respules, Asthalin Respules, Tab Paracetamol, and Syrup Fortiplex.

Given the rapid onset of symmetrical intertriginous eruption following systemic drug exposure, a clinical diagnosis of SDRIFE was made. There was no prior history of reaction to amoxicillin‐clavulanate (clavum). No other differential diagnoses were considered, as the presentation was classical for SDRIFE. Baseline investigations, including Complete Blood Count, Absolute Eosinophil Count, Renal Function Tests, and Liver Function Tests, were within normal limits. No patch testing, biopsy, or histopathology was performed.

The culprit drug was discontinued. The patient was treated with: Tablet Methylprednisolone, Tablet Fexofenadine, topical Clobetasol propionate (cloderm), Emocol‐E cream. The rash began to subside within 15 days, and complete resolution was observed within 14 week.

## 3 Discussion

SDRIFE is a rare cutaneous drug reaction which was first discovered by Andersen et al. in 1984 as baboon syndrome due to erythema of the gluteal region and thigh resembling the red rumps of a baboon [4]. In 2004, Hausermann et al. proposed the term Symmetrical Drug‐related Intertriginous and Flexural Exanthema (SDRIFE) to more accurately reflect the condition's association with systemic drug exposure and its typical distribution pattern [5].

Hauserman et al. outlined 20 criteria to diagnose SDRIFE: (I) exposure to systemic drugs at first or repeated dose (II) erythema of gluteal/perianal area and/or V‐shaped erythema of inguinal/perianal area (III) involvement of at least one other intertriginous or flexural localization (IV) symmetry of affected area (V) absence of systemic signs and symptoms [5].

SDRIFE is caused by the intake of systemic agents at first or repeated exposure [5]. Common medications causing SDRIFE are beta‐lactam antibiotics, especially amoxicillin [6]. However, various drugs like clindamycin, erythromycin, cotrimoxazole, ceftriaxone, nystatin, terbinafine, valacyclovir, and allopurinol have also been reported [5, 6, 7]. The onset of rash could be from hours to 15 days from exposure to the culprit drug [8]. It can affect patients of any age, ranging from 18 months to 84 years [9], and is often misdiagnosed due to its variable presentation.

Although the exact pathogenesis of SDRIFE is still unknown, immune histopathologic findings on skin biopsy have shown CD3+ and CD4+ T‐cells, indicating a possible type IV hypersensitivity reaction [8]. The histopathological features are often non‐specific and may show superficial perivascular infiltration of mononuclear cells with occasional neutrophils and eosinophils [6]. In our case, a biopsy was not performed due to the classical clinical presentation.

Different conditions which can mimic SDRIFE are contact dermatitis, fixed drug eruption, and toxic erythema of chemotherapy [10]. However, the relatively short duration between drug exposure and onset of skin rashes with characteristic findings, as proposed by Hausermann et al., can help differentiate SDRIFE from other similar conditions.

SDRIFE is usually a self‐limiting condition with a good prognosis. Management primarily involves withdrawal of the offending drug and supportive care with topical or systemic corticosteroids and antihistamines. While lesions may begin to resolve within days, full recovery may take up to 3 weeks [11].

In our case, the rapid onset of symmetrical eruption following amoxicillin‐clavulanate exposure, in the absence of mucosal or systemic symptoms, fulfilled all diagnostic criteria for SDRIFE. The classical distribution of lesions with prompt resolution after drug withdrawal further supported the diagnosis. Although palmer and plantar involvement is less commonly reported, it has been documented in a few cases and may represent an extension of the flexural distribution.

## 4 Conclusions

This case is notable for its rare palmar and plantar involvement, a feature described only infrequently in the literature. Given the widespread use of amoxicillin‐clavulanate in clinical practice, it is important to recognize its potential dermatologic adverse effects, including SDRIFE. This benign but striking drug reaction can be easily overlooked without a clear temporal link between drug exposure and the characteristic rash distribution. Clinicians should maintain a high index of suspicion, obtain a thorough drug history, and counsel patients appropriately regarding possible adverse cutaneous reactions.

## Author Contributions

**Niranjan Pudasaini:** conceptualization, resources, supervision, writing – original draft, writing – review and editing. **Anil Kumar Singh Dangol:** conceptualization, resources, supervision, writing – original draft, writing – review and editing. **Surendra Shrestha:** conceptualization, resources, supervision, writing – original draft, writing – review and editing. **Aashutosh Pokharel:** writing – original draft, writing – review and editing. **Aarif Khan:** writing – original draft, writing – review and editing.

## Consent

Authors have obtained informed written patient consent for use of their photographs and medical information to be published online and with the understanding that this information may be publicly available and discoverable via search engines. This consent form is not provided to the Editorial Office but has been retained by the author, and will be provided whenever requested.

## Conflicts of Interest

The authors declare no conflicts of interest.

## Data Availability

Data sharing not applicable to this article as no datasets were generated or analysed during the current study.
